# Supplementary material for: Camu-Camu Fruit Extract Inhibits Oxidative Stress and Inflammatory Responses by Regulating NFAT and Nrf2 Signaling Pathways in High Glucose-Induced Human Keratinocytes
Source: Molecules. 2021 May 26;26(11):3174. doi: 10.3390/molecules26113174 (PMC8198278; doi:10.3390/molecules26113174)
Supplement: Supplementary file 1 [file molecules-26-03174-s001.zip › molecules-1233397-supplementary.pdf]

Table 1. Polymerase chain reaction (PCR) primers used in this experiment.

Gene	Forward	Reverse	Length
GAPDH	ACCACAGTCCATGCCATCAC	CCACCACCCTGTTGCTGTAG	451 bp
TARC/ CCL17	ATGGCCCCACTGAAGATGCT	TGAACACCAACGGTGGAGGT	351 bp
MDC/ CCL22	AGGACAGAGCATGGCTCGCCTACAGA	TAATGGCAGGGAGGTAGGGCTCCTGA	362 bp
RANTES/CCL5	CCCCGTGCCCACATCAAGGAGTATTT	CGTCCAGCCTGGGGAAGGTTTTTGTA	313 bp
IL-8	TCAGTGCATAAAGACATACTCC	TGGCATCTTCACTGATTCTTG	243 bp
